# The Comparison of Intrathecal Morphine and IV Morphine PCA on Pain Control, Patient Satisfaction, Morphine Consumption, and Adverse Effects in Patients Undergoing Reduction Mammoplasty

**Published:** 2015-05-05

**Authors:** Mehtap Karamese, Osman Akdağ, İnci Kara, Gokce Unal Yıldıran, Zekeriya Tosun

**Affiliations:** ^a^Department of Plastic Reconstructive and Aesthetic Surgery, Selcuk University, Konya, Turkey; ^b^Department of Anesthesiology and Reanimation, Selcuk University, Konya, Turkey

**Keywords:** breast reduction, intrathecal morphine, pain control, PCA, patient satisfaction

## Abstract

**Background:** Following breast reduction procedures, the level of postoperative pain can be severe, and sufficient pain control influences a patient's physiological, immunological, and psychological status. **Objective:** The aim of this study was to examine the use of intrathecal morphine (ITM) in breast reduction surgery with patient-controlled analgesia (PCA). **Methods:** Sixty-two female patients who underwent breast reductions with the same technique participated in this study. The study group (ITM + PCA) included 32 patients; a single shot (0.2 mg) of ITM and intravenous morphine with PCA were administered. In the control group, morphine PCA alone was intravenously administered to 30 patients. Comparisons between the groups of cumulative morphine consumption, visual analog scale scores, and patient satisfaction scores, which were the primary outcome measures, and adverse effects, which were the secondary outcome measures, were conducted. **Results:** The patients in the 2 groups had similar degrees of pain and satisfaction scores. The study group had lower cumulative morphine consumption (*P* = .001) than the PCA-only control group; there was no statistically significant difference in adverse effects between the 2 groups. **Conclusion:** Intrathecal morphine may effectively control pain with lower total morphine consumption following breast reduction surgery.

**Level of Evidence:** Level II

Proper pain control following breast surgery can increase patient comfort and satisfaction. Most pain control publications have discussed pain control related to augmentation mammoplasty; however, reduction mammoplasty is associated with pain that is just as intense, due to the requirements for large incisions, extensive dissections, and a pronounced inflammatory response.^1^^-^5 Postoperative pain is often managed with oral and parenteral narcotics. Patient-controlled anesthesia (PCA) is an effective method of pain management that provides the advantages of faster alleviation of pain, better dosage monitoring, and freedom from setting a timer.^6^^,^^7^ However, after breast surgery, some patients still experience pain and PCA-related adverse effects, including sedation, nausea, and vomiting.^8^ Intrathecal opioids have been used for pain management for 2 decades.^6^^,^^9^ It has been shown that the use of intrathecal morphine (ITM) without local anesthesia relieves pain and decreases consumption of systemic morphine in patients with refractory cancer pain.^10^ Although intrathecal opioids have been used widely for postoperative analgesia in a variety of surgical procedures,^11^^,^^12^ there have been no reports assessing the validity of ITM with PCA morphine consumption, pain, or satisfaction in patients undergoing reduction mammoplasty. The purpose of this study was to evaluate the effects of ITM with PCA compared with PCA alone on pain relief, patient satisfaction, morphine consumption, and adverse effects following reduction mammoplasty.

## MATERIALS AND METHODS

### Selection of patients

After institutional review board approval and informed consent were obtained, 62 patients (aged 18 years or older; American Society of Anesthesiologists physical status I–II) who had undergone reduction mammoplasty with the Wise pattern inferior pedicle technique participated in the study. This study was conducted at a single tertiary medical center between January 2010 and December 2014. The exclusion criteria were as follows: patients who required surgery other than the Wise pattern inferior pedicle breast reduction, patients who had a contraindication for spinal intervention or ITM, and patients who were unable to use a PCA pump. Other exclusion criteria included patients with a history of drug abuse or allergy to the study drugs. The day before surgery, the patients were instructed on the use of the PCA device and the method of reporting pain via a visual analog scale (VAS).

A table of random sampling numbers was used to randomly allocated the patients into either the group receiving ITM combined with intravenous PCA (ITM + PCA, *n* = 32) or the group receiving PCA with morphine (PCA, *n* = 30). This study was conducted with approval from the Local Ethics Committee.

### Surgical procedure and administration of ITM

All of the patients underwent the Wise pattern inferior pedicle breast reduction surgery. The surgical procedures have been described previously.^12^All reduction mammoplasty procedures were performed by the same surgeon; the procedure was performed with scalpels and electrocautery. A closed suction drain was used for 1 day. The operative techniques, anesthesia, and monitoring were standardized.

Anesthesia was induced with 2 mg/kg of propofol, 2 μg/kg of fentanyl, and 0.6 mg/kg of rocuronium, administered intravenously. Anesthesia was maintained with a mixture of air (0.5 L/min) and oxygen (0.5 L/min) plus sevoflurane (1%–2%) after tracheal intubation. The same anesthesiologist performed lumbar punctures in the lateral decubitus position at the L_3_–L_4_ or L_4_–L_5_ lumbar vertebral level with a 27G pencil-point spinal needle after anesthesia induction. Patients in the ITM + PCA group received an intrathecal injection of 0.2 mg of morphine. A lumbar puncture was not performed in the PCA-only control group.

On arrival at the postanesthesia care unit, each patient received a PCA pump programmed to deliver an initial morphine bolus of 0.05 mg/kg without base flow if the patient's pain score was higher than 60 on a 100-mm VAS. Upon discharge from the postanesthesia care unit, the pump was reprogrammed to deliver a morphine bolus of 1 mg with an 8-minute lockout without base flow. Patient-controlled anesthesia was maintained during the first 48-hour postoperative period.

### Assessment of pain, patient satisfaction, and adverse effects

A 100-mm VAS was used to evaluate degree of pain and patient satisfaction. The pain scores ranged from 0 mm for *no pain* to 100 mm for the *worst pain imaginable*, and the satisfaction scores ranged from 0 mm for not *satisfied* to 100 mm for *very satisfied*. Degree of pain was documented at 30 minutes, 1, 3, 6, 12, 24, and 48 hours by the same trained plastic surgery assistant who was blinded to the study groups. Patient satisfaction scores were documented after 24 hours and again at 48 hours.

The patients were asked directly if they experienced vomiting, nausea, or pruritus. Intravenous antiemetics and antihistamines (4 mg of ondansetron and 20 mg of diphenhydramine HCl) were administered as rescue drugs at the patient's request. The number of patients with nausea, vomiting, and pruritus was recorded.

Sedation was evaluated on a 5-point scale (0 = fully awake; 1 = sleepy with closed eyes; 2 = asleep but can wake with a simple command; 3 = asleep but can wake with a strong physical command; 4 = cannot be awakened) at 30 minutes, 1, 3, 6, 12, 24, and 48 hours.

Respiratory depression was evaluated using oxygen saturation and respiratory rate. When the degree of oxygen saturation was less than 90% as measured using a pulse oximetry device, and when the respiratory rate was less than 8, the patient was treated with supplemental oxygen and intravenous administration of 0.1 mg of naloxone repeated every 5 minutes until sufficient respiration was restored.

### Statistical analysis

The SPSS statistical package (version 16.0) for Windows (SPSS Inc, Chicago, Ill) was used for statistical analysis. Data are represented as mean ± SD and the number of patients. Data were tested for normality with the Kolmogorov-Smirnov normality test. A χ^2^ test was used for categorical variables. The Student *t* test was used for continuous normally distributed data, and the Mann-Whitney *U* test was used for continuous abnormally distributed data. *P* < .05 was considered statistically significant.

## RESULTS

**Figure 1:**
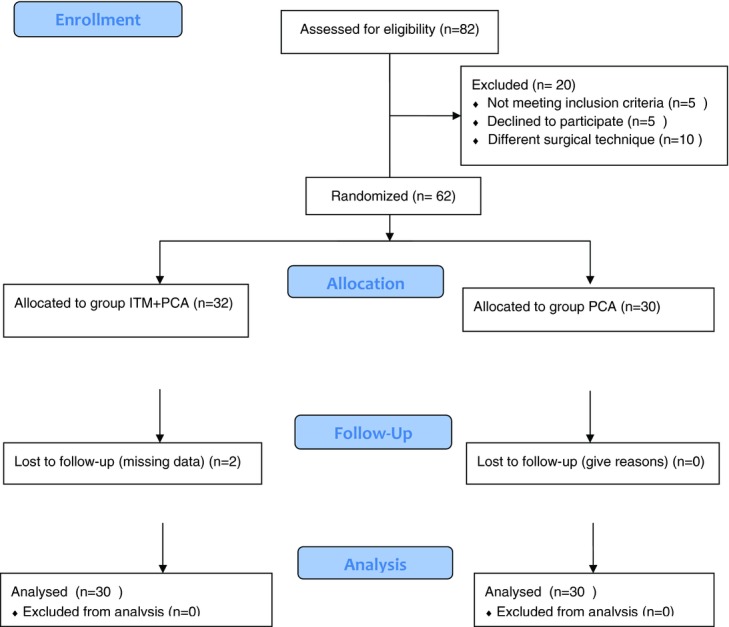
Consort diagram showing the flow of participants through each stage of our randomized trial. ITM indicates intrathecal morphine; PCA, patient-controlled analgesia.

Sixty-two patients were randomly allocated to the 2 groups (Fig 1). Of the patients in the ITM + PCA group, 2 were excluded because of missing data. Thus, a total of 60 patients, aged 24 to 41 years, were compared in terms of cumulative morphine consumption, VAS score, patient satisfaction, sedation scores, oxygen saturation, the number of patients who experienced pruritus or nausea, and the use of antiemetic drugs. The demographic characteristics of the patients are shown in Table 1.

**Figure 2:**
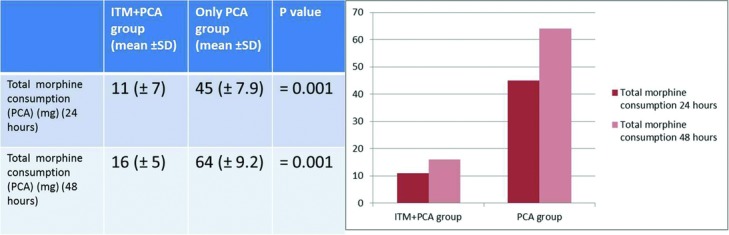
Total morphine consumption in groups. Abbreviations are explained in Figure 1.

The patients who received ITM consumed 11 ± 7 and 16 ± 5 mg of morphine at 24 and 48 hours, respectively. The patients who used PCA alone consumed 45 ± 7.9 and 64 ± 9.2 mg of morphine at 24 and 48 hours, respectively. The differences between the groups were statistically significant (*P* = .001) (Fig 2).

**Figure 3:**
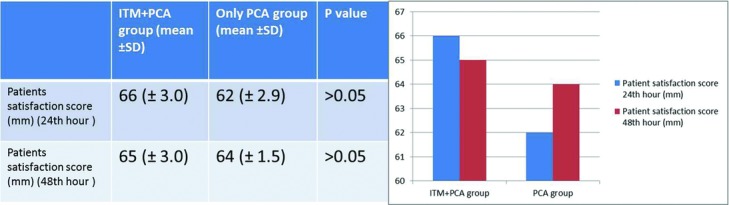
Comparison of patients’ satisfaction scores. Abbreviations are explained in Figure 1.

The patient satisfaction scores at 24 hours were 66 ± 3 and 62 ± 2.9 mm in the PCA-only group and the ITM + PCA group, respectively; at 48 hours, the scores were 65 ± 3 and 64 ± 1.5 mm in the respective groups. There was no statistically significant difference between the 2 groups at either time point (Fig 3).

**Figure 4:**
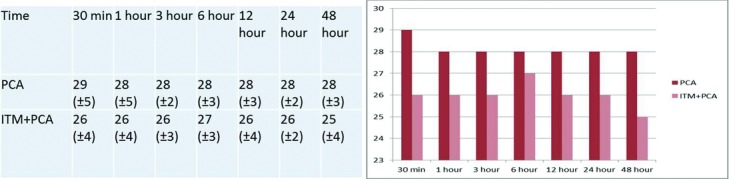
Values of visual analog scale. Abbreviations are explained in Figure 1.

The VAS scores of patients in the ITM + PCA group and the PCA-only group were similar, and there were no statistically significant differences at 30 minutes, 1, 3, 6, 12, 24, or 48 hours (Fig 4).

There were also no statistically significant differences between the 2 groups with respect to nausea, pruritus, or the number of antiemetic drugs used in the postoperative period. Six of the 30 patients in the ITM + PCA group experienced nausea, 2 patients had pruritus, and 1 patient had used antiemetic drugs. Eight of the 30 PCA-only patients experienced nausea, 3 patients had pruritus, and 2 patients had used antiemetic drugs. There were no significant differences between the 2 groups.

The sedation scores, pulse oximetry values, and respiratory rates were comparable in both groups’ at all postoperative periods (Table 2). There was no respiratory depression in any of the patients and no need for naloxone administration. Complications due to the intrathecal intervention, such as postdural puncture headache, were not reported.

## DISCUSSION

This study demonstrated that the analgesic effect of a single ITM injection can reduce cumulative PCA morphine consumption and control acute postoperative pain effectively without differences in any of the adverse effects.

Surgical procedures for breasts vary from reduction to augmentation to breast cancer procedures. Pain control is a major concern among patients undergoing breast surgery. In most pain control studies, the main focus is on breast augmentation procedures.^1^^-^5 However, pain control associated with reduction mammoplasty deserves to be studied because of the long incisions that are used, the extensive dissection area, and the use of liposuction. Adequate pain control after breast operations could reduce the lengths of stay in hospitals. Inadequate pain control following breast reduction procedures influences a patient's physiological, immunological, and psychological status.

Patient-controlled anesthesia with an intravenous opioid, such as morphine, is used effectively for pain management.^6^^,^^7^ It provides pain relief while minimizing adverse effects such as sedation, nausea, vomiting, and pruritus. Morphine does not create specific organ toxicities, such as gastrointestinal bleeding and renal toxicity, unlike acetyl salicylic acid and nonsteroidal anti-inflammatory drugs. Morphine works by binding to receptors both in the central and peripheral nervous systems and in the gastrointestinal tract, and ITM administration directly inhibits spinal opioid receptors. Studies have clearly demonstrated that the spinal bioavailability of hydrophilic drugs (eg, morphine, diamorphine, and hydromorphone) is superior to that of lipophilic opioids (eg, alfentanil, fentanyl, and sufentanil).^9^

Because morphine has many different properties, including greater spinal bioavailability and therefore the ability to be administered neuraxially, it is a suitable choice for treating acute postoperative pain.^14^ However, when using morphine intrathecally, some adverse effects can be expected, which might require decreasing the cumulative morphine dosage. The aims of this study were to reduce cumulative PCA morphine consumption and to control postoperative pain effectively.

Postoperative pain management is one of the greatest challenges for patients who have undergone reduction mammoplasty, and pain control devices tend to cause trouble for patients rather than benefit them. Kryger et al^15^ suggested using a local anesthetic pump for pain control. Rohde et al^16^ presented their experience with pulsed electromagnetic field therapy for pain control after reduction mammoplasty. These studies advocate that patients use a device on the breast. However, it is especially important to control pain without machines, such as a PCA or local anesthetic pump. Intrathecal morphine is effective in this respect. Preoperative usage, efficacy with a single dose, blocking receptors directly without entering the systemic circulation, lower cumulative dosage, and fewer adverse effects are important benefits for encouraging the use of ITM.

The ITM dosages for some surgical procedures have been determined.^9^ In a meta-analysis, a wide range of ITM doses (0.1–4 mg) were investigated in many types of surgical procedures.^17^ According to recent reports, intrathecal administration of 0.2 to 0.4 mg of morphine improves postoperative analgesia without respiratory depression after major surgery.^17^ Therefore, we selected the lowest dose, 0.2 mg, for ITM in the present study. It has also been reported that ITM administration decreases the VAS score up to 24 hours postoperative and reduces opioid requirements up to 48 hours postoperative.^17^ In our study, cumulative morphine consumption was 3 times lower than that with morphine PCA alone.

It is well known that intrathecal administration of morphine causes nausea, vomiting, and pruritus more often than intravenous morphine does.^17^ In our study, fewer patients experienced nausea and pruritus and used antiemetic drugs in the ITM + PCA group than in the PCA-only group. These values were not significantly different between the 2 groups; however, our investigation revealed that cumulative morphine consumption was 3 times higher than morphine consumption in the PCA-only group in our study, which should be taken into account for further randomized controlled studies.

Because morphine can cause sedation and respiratory depression, we monitored our patients closely postoperatively to watch for these adverse effects. There were no significant differences in sedation or oxygen saturation levels between the ITM + PCA and PCA-only groups. When adjusting the morphine dose in PCA, sedation and respiratory sedation dosages should be taken into account.

It has been reported that ITM application does not carry a risk of respiratory depression, and it is used with an internalized intrathecal delivery system to control chronic cancer pain.^10^ However, these are not comparable situations. We used a single shot of ITM to reduce morphine consumption while producing adequate acute postoperative pain management. There is disagreement regarding whether a single injection of neuraxial opioids increases the occurrence of respiratory depression compared with parenteral opioids.^19^ Intrathecal morphine does carry a risk of respiratory depression; therefore, vigilant monitoring should be carried out. In this study, we did not observe any respiratory depression at any of the postoperative time points.

This study had some limitations. While there were no differences in adverse effects between the 2 groups, more adverse effects were experienced in the ITM + PCA group. A possible reason for this finding is that our study did not have sufficient power for secondary outcome measures, such as adverse effects, and we did not perform intrathecal sham interventions in the PCA-only group. Therefore, the study was not double blinded.

## CONCLUSION

We think pain control is an important issue in breast reduction operations. A single-shot ITM was not superior to parenteral narcotics in reducing pain; however, it might be able to relieve pain symptoms with decreased cumulative morphine consumption, which has major implications in developing new strategies for pain management in breast reduction surgery.

## Figures and Tables

**Table 1:** Demographic properties of patients

	ITM + PCA group, mean ± SD	PCA group, mean ± SD	*P*
Age, y	33.8 ± 5	35 ± 4	>.05
Weight, kg	68 ± 6.2	67 ± 5.9	>.05
Excisional tissue, mg	655 ± 73	633 ± 80	>.05

ITM indicates intrathecal morphine; PCA, patient-controlled analgesia.

**Table 2:** Respiratory rates, oxygen saturation, and sedation scores of patients*

	ITM + PCA group (number of patients)		Respiratory rate		Oxygen saturation	
Time	Sedation scores (0/1/2/3/4)	PCA group (number of patients)	ITM + PCA group	PCA group	ITM + PCA group	PCA group
30 min	8/5/2/0/0	7/6/2/0/0	11	10	99	98
1 h	9/5/1/0/0	9/5/1/0/0	11	11	98	98
3 h	12/2/1/0/0	11/3/1/0/0	12	13	97	97
6 h	12/3/0/0/0	12/3/0/0/0	13	14	97	97
12 h	13/2/0/0/0	12/3/0/0/0	11	10	97	97
24 h	14/1/0/0/0	13/2/0/0/0	12	11	98	97
48 h	15/0/0/0/0	15/0/0/0/0	12	12	98	97

*Sedation was evaluated with a 5-point scale (0 = fully awake; 1 = sleepy with closed eyes; 2 = asleep but can wake with a simple command; 3 = asleep but can wake with a strong physical command; 4 = could not be awakened).

Abbreviations are explained in the first footnote to Table 1.
